# New Insights into Upper Tract Urothelial Carcinoma: Lessons Learned from the ROBUUST Collaborative Study

**DOI:** 10.3390/cancers17101668

**Published:** 2025-05-15

**Authors:** Arianna Biasatti, Gabriele Bignante, Francesco Ditonno, Alessandro Veccia, Riccardo Bertolo, Alessandro Antonelli, Randall Lee, Daniel D. Eun, Vitaly Margulis, Firas Abdollah, Takashi Yoshida, Ithaar H. Derweesh, Margaret F. Meagher, Giuseppe Simone, Gabriele Tuderti, Eugenio Bologna, Reza Mehrazin, Soroush Rais-Bahrami, Chandru P. Sundaram, Courtney Yong, Andrea Minervini, Andrea Mari, Luca Lambertini, Matteo Ferro, Nirmish Singla, Savio D. Pandolfo, Daniele Amparore, Enrico Checcucci, Mark L. Gonzalgo, James R. Porter, Alireza Ghoreifi, Roberto Contieri, Sisto Perdonà, Francesco Porpiglia, Hooman Djaladat, Saum Ghodoussipour, Riccardo Autorino

**Affiliations:** 1 Department of Urology, Rush University Medical Center, Chicago, IL 60612, USA; arianna.biasatti@gmail.com (A.B.);; 2 Urologic Clinic, Department of Medicine, Surgery and Health Sciences, University of Trieste, 34149 Trieste, Italy; 3 Division of Urology, Department of Oncology, University of Turin, San Luigi Gonzaga Hospital, 10043 Orbassano, Italy; 4 Department of Urology, Azienda Ospedaliera Universitaria Integrata of Verona, University of Verona, 37126 Verona, Italy; alessandro.veccia@aovr.veneto.it (A.V.);; 5 Fox Chase-Temple Urologic Institute, Philadelphia, PA 19111, USA; 6 Department of Urology, University of Texas Southwestern Medical Center, Dallas, TX 75390, USA; 7 Vattikuti Urology Institute, Henry Ford Hospital, Detroit, MI 48202, USA; 8 Department of Urology and Andrology, Kansai Medical University, Hirakata 573-1191, Osaka, Japan; 9 Department of Urology, UC San Diego School of Medicine, La Jolla, CA 92093, USA; 10 Department of Urology, IRCCS “Regina Elena” National Cancer Institute, 00144 Rome, Italy; 11 Department of Urology, Icahn School of Medicine at Mount Sinai Hospital, New York, NY 10029, USA; 12 Department of Urology, School of Medicine, University of Alabama at Birmingham Heersink, Birmingham, AL 35294, USA; 13 Department of Urology, Indiana University, Indianapolis, IN 47405, USA; 14 Unit of Oncologic Minimally-Invasive Urology and Andrology, Department of Experimental and Clinical Medicine, Careggi University Hospital, University of Florence, 50134 Florence, Italyandrea.mari@unifi.it (A.M.); l.lambertini7@gmail.com (L.L.); 15 Department of Health Science, ASST Santi Paolo and Carlo, University of Milan, 20122 Milan, Italy; 16 Brady Urological Institute, School of Medicine, Johns Hopkins University School of Medicine, Baltimore, MD 21287, USA; 17 Department of Urology, University of L’Aquila, 67100 L’Aquila, Italy; pandolfosavio@gmail.com; 18 Department of Neurosciences and Reproductive Sciences and Odontostomatology, University of Naples Federico II, 80138 Naples, Italy; 19 Department of Surgery, Candiolo Cancer Institute, Fondazione del Piemonte per l’Oncologia, Istituto di Ricovero e Cura a Carattere Scientifico di Candiolo (IRCCS), Candiolo, 10060 Turin, Italy; 20 Desai Sethi Urology Institute, Miller School of Medicine, University of Miami, Miami, FL 33136, USA; 21 Swedish Medical Center, Seattle, WA 98122, USA; 22 Department of Urology, Duke University Medical Centre, Durham, NC 27708, USA; 23 Istituto Nazionale Tumori di Napoli, IRCCS “Fondazione G. Pascale”, 80131 Naples, Italy; 24 Institute of Urology, Kenneth Norris Jr. Comprehensive Cancer Center, University of Southern California, Los Angeles, CA 90089, USA; 25 Division of Urology, Section of Urologic Oncology, Rutgers Cancer Institute, Rutgers Robert Wood Johnson Medical School, New Brunswick, NJ 08901, USA

**Keywords:** Upper Tract Urothelial Carcinoma, ROBUUST registry, ROBUUST 2.0, Urologic Oncology, UTUC

## Abstract

Upper tract urothelial carcinoma (UTUC) is a rare malignancy, representing only 5–10% of urothelial carcinoma. The mainstay of treatment for high-risk patients is radical nephroureterectomy. Given the aggressive behavior of this disease, additional treatments could be required perioperatively in terms of chemotherapy (CHT), either in a neoadjuvant or adjuvant setting. On the other hand, low-risk and selected cases can be managed with kidney-sparing surgery (KSS). The ROBotic surgery for Upper tract Urothelial cancer STudy (ROBUUST) is an ongoing international, multicenter registry of patients undergoing surgery for UTUC. After conducting a literature search in February 2025 using the MEDLINE (via PubMed) and Embase databases, we identified 14 studies based on the ROBUUST data analyses. There are several key topics concerning UTUC that remain under debate and were therefore addressed in these studies, focusing on preoperative evaluation and planning, surgical techniques and intraoperative procedures, additional perioperative treatments, and outcomes. The ROBUUST registry has served as a valuable source for a growing body of investigations focusing on various aspects of UTUC treatment planning, decision-making, and outcomes, providing innovative tools and enabling large-scale, novel analyses.

## Simple Summary

The ROBotic surgery for Upper tract Urothelial cancer STudy (ROBUUST) is an ongoing international, multicenter registry of patients undergoing curative surgery for upper tract urothelial carcinoma (UTUC). Several key topics related to UTUC remain under debate and were therefore addressed in studies derived from ROBUUST data. These studies focus on preoperative planning, surgical approaches, perioperative treatments, and outcomes. The ROBUUST registry has served as a valuable source for a growing body of investigations focusing on various aspects of UTUC, providing innovative tools and enabling large-scale, novel analyses to guide clinical practice and future research.

## 1. Introduction

Urothelial carcinoma (UC) is the second most common urological malignancy in developed countries [1], and 5–10% of cases are accounted for by upper tract urothelial carcinoma (UTUC) [2]. UTUC often presents as an aggressive neoplasm, with over 50% of patients presenting with muscle-invasive disease at diagnosis and high recurrence rates [3]. It occurs approximately two-thirds of the time in the renal pelvis, with the remainder more frequently in the distal ureter [4].

The mainstay of treatment for high-risk patients is radical nephroureterectomy (RNU), but low-risk and selected cases can be managed with kidney-sparing surgery (KSS) [5]. The evolution of surgical techniques and the establishment of robotic surgical platforms in urology have widened the space for debate on UTUC surgical management strategies. Moreover, given the aggressive behavior of this disease, additional treatments could be required. While aiming to enhance survival outcomes, incorporating perioperative chemotherapy (CHT) in the therapeutic process, either in a neoadjuvant (Nad) or adjuvant (Ad) setting, emerges as a viable option and is worthy of scientific discussion [6].

Despite being a relatively rare entity, UTUC poses a considerable clinical challenge due to its diagnostic complexity, biological heterogeneity, and the need for a multidisciplinary therapeutic approach. Advances in diagnostic imaging, medical therapies, and minimally invasive techniques have enriched the therapeutic armamentarium, yet several aspects of management remain controversial.

The ROBotic surgery for Upper tract Urothelial cancer STudy (ROBUUST) is an ongoing international, multicenter registry of patients undergoing curative surgery—either RNU or KSS (segmental ureterectomy or endoscopic ablation)—for UTUC at tertiary referral centers across the United States, Europe, and Asia. After Institutional Review Board approval and a data-sharing agreement at each center, de-identified data were collected and shared among the investigators. The ROBUUST data set includes variables relating to baseline patient features, treatment features (surgical and systemic therapies), treatment outcomes, pathological outcomes, and oncological outcomes.

The aim of this review is to offer a comprehensive analysis of the ongoing challenges in the treatment of UTUC patients and a contemporary summary of the contributions to the recent literature that were possible thanks to this international collaboration.

## 2. Materials and Methods

A literature search was conducted in February 2025 using the MEDLINE (via PubMed) and Embase databases to identify articles published within the last 5 years relative to UTUC, management, and outcomes based on data from the ROBUUST registry. Using the PubMed Advanced Search Builder, queries were performed combining search terms “ROBUUST” and “ROBUUST 2.0” with “Upper Tract Urothelial Carcinoma”. Papers were selected to include only full-length articles in the English language. Congress abstracts were excluded.

Subsequently, the selected articles (Table 1) were thoroughly reviewed, and their findings were analyzed in the context of existing studies and clinical practice guidelines, specifically those from the European Association of Urology (EAU) and the American Urological Association (AUA), with an integrative approach.

## 3. Epidemiology

Smoking has a well-known role among the leading predisposing factors for UTUC [21,22], but mixed results have emerged from several studies analyzing its association and impact on oncological outcomes [23,24,25]. Using ROBUUST data, Bhanvadia et al. [20] executed the largest reported analysis examining cumulative smoking exposure (CSE) on oncologic outcomes for UTUC with the inclusion of modern treatment paradigms involving Nad-CHT and Ad-CHT. They identified key relationships between the burden of smoking exposure and oncologic outcomes. Patients were stratified by levels of CSE and defined as nonsmokers, light exposure (≤19 cigarettes per day and ≤19 years smoking), or heavy exposure (>20 cigarettes per day and >20 years smoking), with all other cases being classified as moderate exposure. Their findings suggest that increasing CSE translates into worse cancer-specific survival (CSS), and that any level of CSE, including light CSE, is associated with worse overall survival (OS) outcomes compared to nonsmokers. Furthermore, smoking cessation did not improve survival outcomes for moderate and heavy smokers.

Furthermore, Zappia et al. [13] conducted an analysis to highlight possible discrepancies in survival outcomes across different racial and ethnic groups with UTUC after RNU. Unlike other genitourinary tract malignancies [26,27,28], this study failed to demonstrate significant differences in survival outcomes when comparing White patients’ outcomes with those of Black, Asian, or Hispanic patients.

## 4. Preoperative Evaluation and Planning

A topic of debate in the management and treatment planning of UTUC is the use of diagnostic ureteroscopy (URS), with or without biopsy, given that some evidence suggests that its use may be associated with an increased risk of intravesical recurrence (IVR) after RNU [29]. European and American guidelines have two different standpoints, with the former recommending the use of diagnostic URS only when imaging and/or voided urine cytology do not provide definitive data for diagnosis and risk stratification [5], while the latter suggests its adoption in patients suspected of having UTUC for any lesion identified on imaging [30]. At the same time, the role of KSS is evolving in patients with high-risk UTUC, given the growing body of studies assessing the relative comparability of oncologic outcomes with RNU [17,31,32].

URS may play a pivotal role as a potential decision-making tool for the most appropriate treatment between RNU and KSS. A study by Ditonno et al. [12], based on ROBUUST registry data, aimed to evaluate its prognostic role in high-risk UTUC patients.

The cohort was stratified into two groups based on whether a diagnostic URS was performed or not. Patients with a higher American Society of Anesthesiologists (ASA) score were more likely to undergo URS (OR 2.07, 95% CI 1.56–2.75). This may be attributed to the pursuit of a more precise diagnosis, aiming to spare more fragile individuals from invasive procedures. Conversely, patients with a tumor ≥2 cm (OR 0.49, 95%CI 0.35–0.68) and at a clinically locally advanced stage (OR 0.33, 95%CI 0.23–0.46) were significantly less likely to undergo diagnostic URS before treatment and were therefore recommended directly for RNU. With high-quality biopsies (only 4.5% were non-diagnostic) and a high concordance rate (80.1%) in terms of tumor grade with the final surgical pathology, this study supports the effectiveness of diagnostic URS as a valuable tool in aiding diagnosis and treatment decision-making, especially when performed at high-volume centers. The survival analysis revealed a significant advantage in terms of OS and CSS among patients undergoing preoperative URS and biopsy, suggesting a better selection of candidates for curative surgical treatment following the acquisition of biopsy-related information.

Renal function is a key topic when discussing UTUC and its management. The two principal therapeutic tools, RNU and cisplatin-based CHT, are indeed strongly related to it, as the surgical removal of a renal unit often results in significant renal function impairment [33,34], and as the use of a cisplatin CHT regimen is not recommended when the estimated glomerular filtration rate (eGFR) drops below 50–60 mL/min/1.73 m^2^ [5,35]. Therefore, a thorough preliminary patient assessment is fundamental.

**Figure 1:**
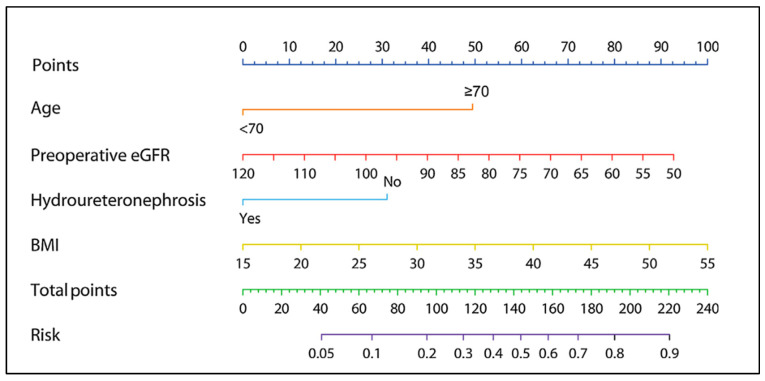
Nomogram for the prediction of postoperative eGFR < 50 mL/min/1.73 m^2^ after RNU based on a multivariable model. Instructions for utilization: locate the patient’s preoperative eGFR on the corresponding axis. Draw a line straight downward to the point axis to determine how many points toward the probability of postoperative eGFR < 50 mL/min/1.73 m^2^ the patient receives for the preoperative eGFR. Repeat the process for each additional predictor. Add the points for each of the variables. Locate the final sum on the total points axis. Draw a line straight up to find the patient’s risk probability. BMI: body mass index; eGFR: estimated glomerular filtration rate; RNU: radical nephroureterectomy. Reproduced from Wu et al. [10], *European Urology Focus*, 2022; with permission granted by Elsevier.

In this scenario, an innovative evaluation model was designed by Wu et al. [10] using data from the ROBUUST registry. The model incorporated age, body mass index (BMI), preoperative eGFR, and hydroureteronephrosis. A cut-off of eGFR <50 mL/min/1.73 m^2^ at 3 months after surgery was set for cisplatin ineligibility. They developed a nomogram to predict renal function insufficiency in patients undergoing cisplatin-based adjuvant chemotherapy after minimally invasive RNU (Figure 1). The model successfully stratified patients into low- and high-risk categories for developing impaired renal function postoperatively. The clinical usefulness of this prediction tool is especially evident when it comes to decision-making and treatment planning. Specifically, it can help select patients at a lower risk of cisplatin ineligibility who are favorable surgical candidates and could potentially skip neoadjuvant chemotherapy. On the other hand, it can identify those patients who are at a high risk of renal insufficiency postoperatively and who would probably benefit from neoadjuvant chemotherapy.

In the preoperative context and planning, another ROBUUST-based study gave important feedback on decision-making in referral to Nad-CHT. There are several reasons to shed light on the neoadjuvant setting in the management of UTUC. Firstly, UTUC shares biological, histological, and molecular similarities with bladder UC, where Nad-CHT has become a standard of care, thanks to significant improvements in overall OS. Secondly, UTUC is associated with a high risk of lymph node (LN) involvement and distant metastasis at the time of diagnosis [36], addressing the need for systemic therapy to target micro-metastatic disease, especially in advanced stages.

Using the ROBUUST registry, Tuderti et al. [14] conducted the largest real-world data retrospective investigation to date, comparing survival outcomes between patients with UTUC who received RNU only, Nad-CHT, or Ad-CHT. Their results showed that, in specific scenarios such as locally advanced and clinically positive LN disease (cN+), Nad-CHT seems to offer a significant benefit in terms of CSS and OS, with a negligible impact on surgical morbidity. These findings could be a possible stimulus for future trials focusing on advanced disease stages.

From the aforementioned studies emerges the growing emphasis that has been given to renal function and the pursuit of its preservation, either with nephron-sparing surgeries or balanced systemic treatments. The clinical importance of preserving renal function has been highlighted in a study by Puri et al. [15], in which the association between postoperative functional decline and survival outcomes was evaluated. The novel finding from their investigation was that surgically induced development of chronic kidney disease with an eGFR <45 mL/min/1.73 m^2^ (defined as CKD-S3b) was associated with worse all-cause mortality (ACM) (HR 1.42, *p* = 0.032), but that a decline to eGFR <60 mL/min/1.73 m^2^ (CKD-S3a) was not, defining an innovative threshold. Increasing age at diagnosis (OR 1.04, *p* = 0.029) and decreasing preoperative eGFR (OR 1.01, *p* < 0.001) were identified as predictors of the development of de novo CKD-S3b. Notably, the receipt of Nad-CHT (OR 2.07, *p* = 0.006) and Ad-CHT (OR 1.41, *p* = 0.012) was associated with a higher risk of developing CKD-S3b. In this analysis, >70% of patients with UTUC undergoing RNU developed postoperative CKD-S (34.7% CKD-S3a and 39.6% CKD-S3b), with those experiencing CKD-S3b being at an increased risk of aggravated ACM. These findings call for the exploration of non-nephrotoxic systemic therapy and the prioritization of nephron-preserving management strategies when feasible and oncologically safe.

## 5. Surgical Techniques and Innovations

### 5.1. Single Stage Xi^®^ Robot-Assisted Nephroureterectomy (RANU)

With the increasing role of robotic surgery in the management of genitourinary cancers [37], evidence on curative robotic surgery for UTUC has been growing over the past decade; however, it was sparse and of low quality [38].

An accurate report of the surgical steps and a detailed description of the technique of single-stage RANU using the Da Vinci Xi^®^ surgical system (Intuitive Surgical^®^, Sunnyvale, CA, USA) was reported by Veccia et al. [9]. First, it was highlighted how the robotic platform facilitates the “multi-quadrant” approach that is required for this procedure. Indeed, the port placement in a straight-line configuration, also described by Patel et al. [39], was validated as a key point in performing a seamless “single-stage” RANU, which does not require patient repositioning or robot re-docking. Moreover, it maximizes freedom of movement and provides good ergonomics during critical steps of the procedure, such as lymphadenectomy and management of the bladder cuff. Specifically, regarding the latter surgical step, the goal is to perform an en bloc excision of the distal ureter, vesico-ureteral junction, and bladder cuff. Furthermore, the assessment of surgical outcomes in terms of operative time, estimated blood loss, complication rate, and length of stay confirmed that single-stage Xi^®^-RANU is a safe, reproducible, and effective minimally invasive procedure for the treatment of UTUC.

**Figure 2:**
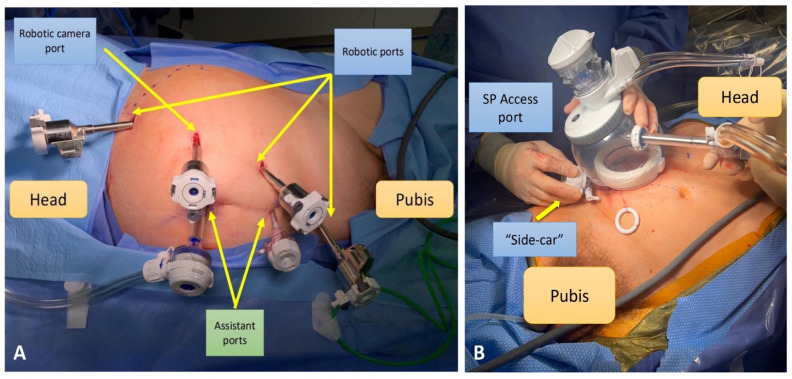
(**A**) Port placement for Xi^®^ single-stage robot-assisted nephroureterectomy (RANU) with bladder cuff excision; (**B**) the single-port (SP) access port and “side-car” placement for RANU.

In the future, a role in this space might be gained by single-port robotic surgery. Only a few reports on single-port RANU have been reported to date [40], and the main advantage seems to be that it is able to perform the procedure in a single stage with retroperitoneal access. The ROBUUST collaborative group is working on a multi-institutional analysis of the procedure (Figure 2).

### 5.2. Laparoscopic RNU (LRNU) vs. RANU

LRNU and RANU were compared by Veccia et al. [41] in a propensity score-matched pair analysis. Moreover, the concept of a “tetrafecta” outcome was introduced as a proxy of “surgical quality”, defined as the concomitant occurrence of bladder cuff excision, lymph node dissection (LND), no complications, and negative surgical margins.

In general, robotic and laparoscopic techniques offered comparable outcomes, with the laparoscopic approach having a higher rate of overall complications (although related to low-grade events of limited clinical relevance) and a longer length of stay.

A technical advantage was highlighted in this study regarding the management of the bladder cuff. The multi-quadrant nature of RNU makes intracorporeal bladder cuff excision difficult during LRNU [42]. On the other hand, the maneuverability of the robotic instruments permits higher versatility and easier dissection of the distal ureter [37] as also shown in this study, in which a significantly higher rate of bladder cuff excision was described in the RANU group (*p* < 0.001).

### 5.3. Bladder Cuff Excision

Bladder cuff excision is defined as the complete removal of the intramural ureter along with a portion of the bladder wall surrounding the ureteral orifice, performed during RNU to minimize the risk of local recurrence at the distal ureteral stump [43].

Indeed, being able to standardize and perform bladder cuff excision on a regular basis is fundamental from an oncological point of view. The European Association of Urology (EAU) guidelines have recommended the addition of a bladder cuff to RNU since 2004, and have continued to recommend it in the most recent 2023 guidelines [5,44].

Accordingly, using the ROBUUST database, Yong et al. [16] demonstrated that bladder cuff excision improves recurrence-free survival (RFS), regardless of the surgical technique used. More specifically, the excision of the bladder cuff conferred a decreased risk of bladder-specific recurrence, compared with no excision. Formal excision, pluck, stripping, or intussusception technique are valid if the intramural ureter and ureteral orifice are excised. The omission of bladder cuff excision, on the other hand, has been consistently associated with higher rates of IVR, emphasizing its central role in oncological safety.

Interestingly, the study showed that while all excision methods reduced bladder recurrence, they did not significantly impact metastasis-free survival (MFS), OS, or CSS.

### 5.4. Lymphadenectomy

Studies assessing the value of LND in RNU on oncologic outcomes have been controversial [45,46]. EAU guidelines recommend LND for the optimal tumor staging in clinical circumstances suspicious for LN positivity, but the impact of LND in circumstances of clinical node negativity is unclear [47,48].

The large scale of the ROBUUST database allowed a thorough analysis of this surgical step and its outcomes [8]. The robotic cohort comprised patients who did not undergo LND (pNx), patients who underwent LND with negative nodes (pN0), and patients who underwent LND with positive nodes (pN+). First, the performance of LND was not associated with an increased incidence of intraoperative or postoperative complications, suggesting that the robotic approach is a safe setting for this procedure. Second, while LND was not associated with cancer-specific and OS benefit in the setting of pathological node-negative disease, it may provide important prognostic information. Indeed, high tumor grade and increasing tumor size were identified as independent predictors of pN+ disease, highlighting a possible unique delineation of selection criteria for LND in the setting of clinical node-negative (cN0) disease. Furthermore, LN yields ≥10 in cN0 patients may be associated with improved RFS.

### 5.5. Distal Ureterectomy

A direct comparison of patients undergoing either robot-assisted distal ureterectomy (RADU) or RANU for high-risk distal ureter tumors was possible thanks to the international and multi-institutional cohort of the ROBUUST database [17]. Across various guideline bodies, some still recommend avoiding the use of KSS in high-risk UTUC patients, except for mandatory indications, such as solitary kidney, and in highly selected cases with a relative contraindication to RNU [49]. In contrast, others have conformed to the increasing body of evidence that demonstrates promising outcomes with DU for primary definitive surgical treatment of distal ureteral tumors [30,50]. This analysis by Ditonno et al. [17] showed comparable outcomes in terms of RFS, MFS, and OS between RADU and RANU. Therefore, the ongoing challenge lies now in identifying the prognostic factors that effectively predict worse oncologic outcomes among high-risk patients eligible for KSS. According to the aforementioned study, tumor grade, stage, and treatment modality were not identified as negative prognostic factors. In contrast, the presence of hydronephrosis and previous radical cystectomy was associated with adverse outcomes. Notably, an advantage in terms of preservation of postoperative renal function and a lower incidence of postoperative complications among RADU patients was observed. These findings altogether suggest that KSS should be considered as a suitable option for selected patients with high-risk distal ureteral UTUC.

## 6. Outcomes

### 6.1. Mid-Term Outcomes of RANU

The largest series of RANU patients was obtained from the ROBUUST database. The analysis by Ditonno et al. [18] sought to evaluate the surgical, functional, and mid-term oncological outcomes of over 1100 patients. The safety and feasibility of RANU were sustained by the extremely low rates of intraoperative and major 30-day postoperative complications (2.7% and 3.7%, respectively). Moreover, the administration of Nad-CHT was not a significant predictor of 30-day postoperative complications, and the rate of positive surgical margins (PSM) was very low (4.7%). Regarding the oncological outcomes, they reported a 3-year RFS of 59% and a 3-year MFS of 76%. The 3-year OS and CSS were 76% and 88%, respectively. They also conducted a sub-analysis on patients with locally advanced disease, noting that they experienced a 3-year RFS of 53% and a 3-year MFS of 54%. The 3-year OS and CSS rates were 70% and 76%, respectively. However, in this context, oncological outcomes appear to be primarily driven by disease-specific factors rather than the surgical approach [3,51]. The execution of bladder cuff excision emerged as a significant predictor of RFS in the multivariate analysis, and the most common approach to bladder cuff excision found in this series was robot-assisted extravesical excision. Overall, this study contributed to the growing body of evidence supporting the increasing adoption of RANU in diverse clinical settings.

### 6.2. Intravesical Recurrence

Indeed, a transition to minimally invasive techniques in urological surgery has occurred over the past decade [52]. However, regardless of the surgical technique used, 22% to 47% of patients with UTUC will experience IVR [53]. Noting the persistence of the relevance of this event, Katims et al. [7] aimed to identify risk factors for IVR in a completely minimally invasive (laparoscopic or robotic) RNU cohort, using the multi-institutional ROBUUST registry. In this analysis, the rate of IVR was 22.7%, consistent with previous studies [54]. Interestingly, they identified the execution of preoperative ureteroscopic biopsy and bladder cuff management with transurethral resection as key risk factors for IVR. More specifically, transurethral resection of the bladder cuff, probably due to incomplete resection or tumor spillage, increased the risk for IVR by 95% and had a nearly threefold increased risk on multiple regression. Furthermore, detection of PSM was identified among treatment-specific risk factors for IVR. Therefore, while aiming for oncological success in patients with UTUC, it is critical and fundamental to identify and mitigate risk factors for IVR.

**Figure 3:**
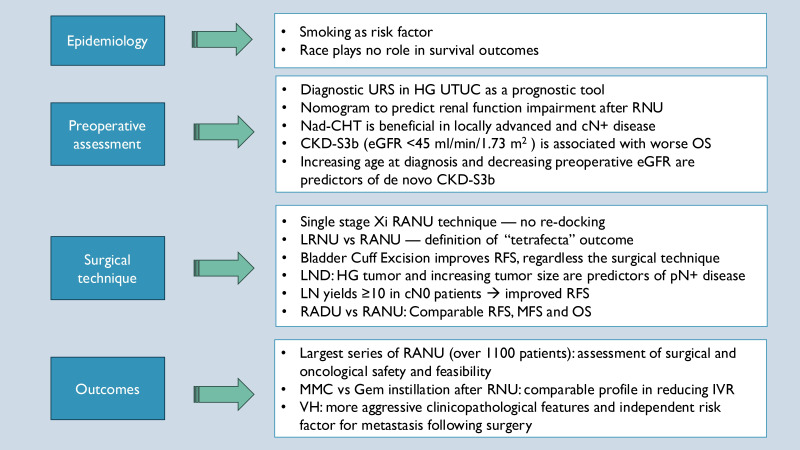
Key points on UTUC from studies based on ROBUUST registry data analysis. **Abbreviations:** URS: ureteroscopy; HG: high grade; UTUC: upper tract urothelial carcinoma; RNU: radical nephreureterectomy; Nad-CHT: neoadjuvant chemotherapy; CKD-S3b: surgically induced chronic kidney disease; OS: overall survival; RANU: robot-assisted nephreureterectomy; LRNU: laparoscopic radical nephreureterectomy; RFS: recurrence-free survival; LND: lymph node dissection; pN+: pathologically positive lymph node disease; RADU: robot-assisted distal ureterectomy; MFS: metastasis-free survival; MMC: mitomycin C; Gem: gemcitabine; IVR: intravesical recurrence; VH: variant histology.

Relative to IVR, the most recent ROBUUST registry-based study is the first direct comparative analysis evaluating the efficacy of single chemotherapy instillation of mitomycin C (MMC) and gemcitabine (Gem), administered within 7 days after minimally invasive RNU for UTUC [51]. The primary objective of this analysis was to discern any potential differences in IVR rates and bladder recurrence-free survival (bRFS) between these two therapeutic adjuvant strategies. Indeed, while the EAU and the National Comprehensive Cancer Network (NCCN) guidelines recognize the benefit of postoperative bladder instillation, they refrain from favoring any specific chemotherapeutic agent due to the current lack of conclusive comparative evidence [5,44]. The comparison between IVR rates of the MMC and Gem group did not reach conventional levels of statistical significance. The Kaplan–Meier survival analysis, on the other hand, suggested an early divergence in the treatment effect within the initial 12 months after RNU and post-operative bladder instillation. Despite this early trend, the long-term analysis over 36 months did not substantiate a significant disparity in terms of bRFS (Figure 3). This study thus provides an initial perspective on the comparative efficacy of single instillation after RNU between MMC and Gem, demonstrating a comparable profile in reducing IVR between the two agents. If these results were to be confirmed, the equivalent efficacy of the two drugs could offer new perspectives from a cost-management standpoint, given that Gem is significantly less expensive than MMC [52].

### 6.3. Variant Histology

Finally, also from a histopathological standpoint, the ROBUUST database was a crucial source of information. Douglawi et al. [11] sought to investigate the impact of variant histology (VH) on oncologic outcomes, compared to pure UC, in a large multicenter cohort of patients with UTUC treated with RNU. VH was found in approximately 10% of patients, and it showed the presence of more aggressive clinicopathological features. Indeed, an association of VH with a higher clinical stage at diagnosis, higher pathologic grade and stage, positive surgical margin rate, and lymphovascular invasion was noted. These findings also resulted in a higher need for adjuvant chemotherapy in this group of patients. Moreover, the presence of VH was identified as an independent risk factor for future metastasis following surgery, together with pathologic T stage and positive surgical margins.

The results of this study are important for patient risk stratification and perioperative counseling for appropriate decision making.

## 7. Strengths and Limitations

This review benefits from the inclusion of data derived from a large, international, multicenter collaboration, which enhances its relevance and scope. Figure 3 provides a visual summary of the key findings across the ROBUUST-derived studies, highlighting consistent clinical messages and strengths. Nonetheless, we acknowledge certain limitations. First, the review is inherently subject to selection bias, as it focuses exclusively on studies produced within the ROBUUST registry and may therefore not reflect the full breadth of the existing literature on UTUC. Moreover, while we aimed to capture the most impactful contributions from the registry, some important aspects of UTUC management remain unexplored due to a lack of published data, such as the availability of long-term follow-up outcomes and the role of Bacillus Calmette–Guérin instillation.

## 8. Conclusions

UTUC is a rare but aggressive disease that needs to be considered in every aspect, from the moment of diagnosis to treatment planning and decision-making. The ROBUUST registry has proven to be a valuable source of data, enabling thorough analyses due to its large scale. Several key topics related to the treatments and outcomes of UTUC have been addressed, and diverse insights have been highlighted, which can serve as a foundation for current clinical practice while simultaneously stimulating future studies and continuous advancement in this field. The ROBUUST registry has become an essential resource for a growing body of investigations into UTUC, providing innovative tools and enabling large-scale novel analyses to guide clinical practice and future research.

## Figures and Tables

**Table 1:** Summary of the published studies based on ROBUUST-derived data.

Author, Year	Patients, n	Aim of the Study	Main Findings
Katims et al., 2021 [7]	485	To assess risk factors for IVR after minimally invasive RNU	Preoperative ureteroscopic biopsy, transurethral resection of bladder cuff and PSM are independent risk factors for IVR
Hakimi et al., 2022 [8]	877	To evaluate outcomes of LND after RANU	LND not associated with intra or postoperative complications; HG tumor and tumor size predictors of pN+ disease
Veccia et al., 2022 [9]	148	To report the surgical steps and technique of single stage Xi RANU	Xi Robotic platform facilitates multi-quadrant approach without need for patient repositioning or robot re-docking
Wu et al., 2022 [10]	490	To predict CKD after RNU	A nomogram based on age, BMI, preoperative eGFR and HUN can help stratify patients in low and high risk of post-RNU CKD
Douglawi et al., 2023 [11]	687	To investigate the impact of VH on oncologic outcomes	VH found in 10% of patients after RNU and associated with risk of future metastasis
Ditonno et al., 2024 [12]	1912	To evaluate prognostic role of diagnostic URS in high-risk UTUC	Significant advantage in OS and CSS if URS and biopsy are performed
Zappia et al., 2024 [13]	1446	To highlight differences in survival outcomes across racial groups after RNU for UTUC	No significant differences when comparing White, Black, Asian, or Hispanic patients
Tuderti et al., 2024 [14]	1994	To compare survival outcomes between patients with UTUC who received RNU only, Nad-CHT or Ad-CHT	In locally advanced and cN+ disease, Nad-CHT offers better CSS and OS
Puri et al., 2024 [15]	1862	To evaluate the association between CKD-S after RNU and survival outcomes	CKD-S3b is associated with worse ACM
Yong et al., 2024 [16]	1718	To determine whether bladder cuff excision and its technique influence survival outcomes	Bladder cuff excision improves RFS, regardless the surgical technique
Ditonno et al., 2024 [17]	477	To directly compare the outcomes of RADU and RANU for high-risk distal ureter tumors	Comparable outcomes in terms of RFS, MFS and OS between RADU and RANU
Ditonno et al., 2024 [18]	1118	To evaluate surgical, functional, and mid-term oncological outcomes after RANU	Postoperative complications: 14.1%; PSM: 4.7%; At 3-years: RFS 59%, MFS 76%, OS 76%, CSS 88%
Bologna et al., 2025 [19]	377	To compare efficacy of MMC and Gem instillation within 7 days after RNU	No difference in IVR and bRFS rates between MMC and Gem instillation
Bhanvadia et al., 2025 [20]	1730	To examine impact of CSE on oncologic outcomes for UTUC	Increasing CSE translates into worse CSS.Any level CSE corresponds to worse OS

**Abbreviations**: IVR: intravesical recurrence; RNU: radical nephroureterectomy; PSM: positive surgical margins; LND: lymph node dissection; RANU: robot-assisted nephroureterectomy; HG: high grade; pN+: pathologically positive lymph node disease; URS: ureteroscopy; UTUC: upper tract urothelial carcinoma; Ad-CHT: adjuvant chemotherapy; CSS: cancer-specific survival; OS: overall survival; CKD: chronic kidney disease; BMI: body mass index; eGFR: estimated glomerular filtration rate; HUN: hydroureteronephrosis; VH: variant histology; Nad-CHT: neoadjuvant chemotherapy; CKD-S3b: surgically induced chronic kidney disease (eGFR < 45 mL/min/1.73 m^2^); ACM: all-cause mortality; RFS: recurrence-free survival; RADU: robot-assisted distal ureterectomy; MFS: metastasis-free survival; MMC: mitomycin C; Gem: gemcitabine; CSE: cumulative smoking exposure.

## Abbreviations

URS	Ureteroscopy
HG	High Grade
UTUC	Upper Tract Urothelial Carcinoma
RNU	Radical Nephreureterectomy
Nad-CHT	Neoadjuvant Chemotherapy
Ad-CHT	Adjuvant Chemotherapy
cN+	Clinically Positive Lymph Node Disease
CKD-S	Surgically Induced Chronic Kidney Disease
OS	Overall Survival
RANU	Robot-Assisted Nephroureterectomy
LRNU	Laparoscopic Radical Nephroureterectomy
RFS	Recurrence Free Survival
LND	Lymph Node Dissection
pN+	Pathologically Positive Lymph Node Disease
LN	Lymph Node
cN0	Clinically Negative Lymph Node Disease
RADU	Robot-Assisted Distal Ureterectomy
MFS	Metastasis-Free Survival
MMC	Mitomycin C
Gem	Gemcitabine
IVR	Intravesical Recurrence
VH	Variant Histology
